# Inhibition of Lipolysis in the Novel Transgenic Quail Model Overexpressing G_0_/G_1_ Switch Gene 2 in the Adipose Tissue during Feed Restriction

**DOI:** 10.1371/journal.pone.0100905

**Published:** 2014-06-25

**Authors:** Sangsu Shin, Young Min Choi, Jae Yong Han, Kichoon Lee

**Affiliations:** 1 Department of Animal Sciences, The Ohio State University, Columbus, Ohio, United States of America; 2 World Class University Biomodulation Major, Department of Agricultural Biotechnology, Research Institute for Agriculture and Life Sciences, College of Agriculture and Life Sciences, Seoul National University, Seoul, Korea; University of Cordoba, Spain

## Abstract

In addition to the issue of obesity in humans, the production of low-fat meat from domestic animals is important in the agricultural industry to satisfy consumer demand. Understanding the regulation of lipolysis in adipose tissue could advance our knowledge to potentially solve both issues. Although the G_0_/G_1_ switch gene 2 (G0S2) was recently identified as an inhibitor of adipose triglyceride lipase (ATGL) *in vitro*, its role *in vivo* has not been fully clarified. This study was conducted to investigate the role of G0S2 gene *in vivo* by using two independent transgenic quail lines during different energy conditions. Unexpectedly, G0S2 overexpression had a negligible effect on plasma NEFA concentration, fat cell size and fat pad weight under *ad libitum* feeding condition when adipose lipolytic activity is minimal. A two-week feed restriction in non-transgenic quail expectedly caused increased plasma NEFA concentration and dramatically reduced fat cell size and fat pad weight. Contrary, G0S2 overexpression under a feed restriction resulted in a significantly less elevation of plasma NEFA concentration and smaller reductions in fat pad weights and fat cell size compared to non-transgenic quail, demonstrating inhibition of lipolysis and resistance to loss of fat by G0S2. Excessive G0S2 inhibits lipolysis *in vivo* during active lipolytic conditions, such as food restriction and fasting, suggesting G0S2 as a potential target for treatment of obesity. In addition, transgenic quail are novel models for studying lipid metabolism and mechanisms of obesity.

## Introduction

Obesity is considered a major health problem in an increasing number of countries and is underscored by adiposity. Adiposity collectively refers to fat cell numbers and sizes in the body and is regulated by developmental, nutritional, and hormonal signals. Major health issues closely associated with obesity include diabetes, hypertension, and cardiovascular diseases. In adult animals, including humans, changes in adipose tissue mass mainly result from plasticity of adipocyte sizes rather than from changes in cell numbers. Mature adipocytes are composed of 70 - 90% triacylglycerides (TAG) that are synthesized and hydrolyzed in response to energy status, regulating adipocyte size and consequently affecting fat mass. In addition, it is important to produce low-fat meat in the agricultural industry as providing a high-quality meat is a possible approach to reducing obesity.

Recent discoveries in the initial process of lipolysis to hydrolyze TAG into a fatty acid and diacylglycerol (DAG) revealed a complicated regulation of adipocyte size [1]–[3]. Adipose triglyceride lipase (ATGL, also named patatin-like phospholipase domain containing protein 2) is a recently discovered TAG hydrolase, which is considered a major initiator of lipolysis in adipose tissues [1], [4]–[7]. ATGL is mainly expressed in adipose tissues, and a deficiency of ATGL in mice causes excessive accumulation of fat [8]. ATGL is composed of a patatin domain, which has an active site for its enzyme activity, and a hydrophobic domain for binding to lipid droplets (LD) required for ATGL enzyme activity [2], [9]–[11]. There are two major regulators of ATGL activity identified to date. Activation of ATGL requires comparative gene identification-58 (CGI-58), which recruits ATGL to the LD by binding directly to the amino acid sequence within the patatin domain of ATGL [2], [11], [12]. Another regulator of ATGL is the G_0_/G_1_ switch gene 2 (G0S2), which was recently identified as an inhibitor of ATGL in 3T3-L1 adipocytes [3]. Comparative analysis of G0S2 amino acid sequences among animals and humans revealed a conservation of the hydrophobic domain, which is required for binding directly to the patatin domain of ATGL [3], [13], [14]. Similar to ATGL, G0S2 expression is mainly found in adipose tissues and is greatly up-regulated during preadipocyte differentiation in humans, mice, pigs, and avian species [3], [13]–[17]. In adipose tissues, G0S2 is more highly expressed in mature fat cells containing TAG than in preadipocytes, which suggests that G0S2 is closely related to fat metabolism. The expression of G0S2 in adipose tissues is also regulated by feeding conditions. Feeding conditions promote up-regulation while down-regulation occurs during fasting, further supporting the inhibitory role of G0S2 in ATGL-mediated lipolysis [13], [14], [17]. Nutrition-dependent expression pattern of G0S2 usually displays an inverse relationship to ATGL expression; however, these findings need to be further validated to provide direct evidence for the inhibitory function of G0S2 *in vivo*.

Herein, we produced transgenic quail that overexpressed the G0S2 gene in adipose tissues to investigate the role of G0S2 *in vivo*. To test whether the exogenous G0S2 could affect fat metabolism depending on the energy conditions, quail were subjected to an *ad libitum* feeding condition, when the amounts of ATGL expression are very low and endogenous G0S2 expression is very high, as well as a feed restriction condition, when the amounts of ATGL expression are very high and G0S2 expression is low.

## Materials and Methods

### Experimental quail and ethics statement

Japanese quail (*Coturnix coturnix japonica*) used in this study were maintained in the poultry center at The Ohio State University (OSU). The quail were always allowed to access feed *ad libitum*, except in the feed restriction study. For feed restriction that ran in parallel with *ad libitum* feeding, each quail was fed *ad libitum* until 6 weeks of age and then restricted to 12 g (about 60% of daily normal intake) of feed per day for 2 weeks. To investigate the role of G0S2 under the various feeding conditions, five to eight male quail hatched from the same parents were used for each non-transgenic or transgenic group for both lines. For the fasting experiment, all four groups of quail were fasted for 4 or 12 hours at 7 weeks of age. All of the experimental procedures were conducted in accordance with protocols approved by the Institutional Animal Care and Use Committee (IACUC) of OSU and the Prevention of Cruelty to Animals Act (1986).

### Vector construction

The chicken G0S2 gene was amplified from cDNA of chicken adipose tissue by PCR with forward, 5′- GCCTCAGAACGGAGCTCTTCCT -3′, and reverse, 5′- GCAGCAGAGCCGTGTGCT -3′, primers and cloned into the pCR2.1 (Invitrogen, Carlsbad, CA, USA) cloning vector [14]. For the adipose tissue-specific promoter, the 2 kb upstream promoter region of the chicken fatty acid binding protein 4 (cFABP4) gene was amplified from chicken genomic DNA by PCR with forward, 5′-CAGGTGCTTCAACTCTCTCCCAA-3′, and reverse, 5′-CAGGCCAGGTAGCAGTCTTTCAC-3′, primers and cloned into the pCR2.1 vector. To generate the pLT-cFABP4-cG0S2 lentiviral vector, cFABP4 promoter and cG0S2 gene were replaced to sites of the RSV promoter and green fluorescence protein gene, respectively, of pLTReGW vector described in our previous report [18].

### Production and concentration of lentivirus

The lentivirus was produced by the methods used by Shin et al. [18]. Briefly, one third of the 95% confluent 293FT cells (Invitrogen) in a 100 mm culture dish were seeded one day before transfection, fed with fresh medium two hours before transfection, and co-transfected with 10 µg of transfer vector (pLT-cFABP4-cG0S2) and 10 µg of packaging mixture (pLP1, pLP2, and pLP/VSVG; Invitrogen) using calcium-phosphate co-precipitation for 6 hours. The medium was replaced with 5 ml fresh culture medium. After 2 days of transfection, the medium containing the virus was harvested, filtrated, and concentrated to 100 X by ultracentrifugation (Beckman Coulter, Inc., Brea, CA, USA) at 26,000 rpm for 2 hours.

### Production of the founder quails

Fresh eggs of wild-type Japanese quail were cleaned and sanitized with 70% ethanol and placed laterally on a tray for 4 hours. After sanitizing the eggs again with 70% ethanol, a small window about 5 mm in diameter was made on the lateral apex of the eggshell using fine-tip tweezers. Then, 1 to 2 µl of concentrated virus was injected into the subgerminal cavity by using the microinjection system (Tritech Research, Inc., Los Angeles, CA, USA) under a stereomicroscope (Olympus America Inc., Center Valley, PA, USA). After sealing the window with paraffin film, the eggs were incubated for 14 days at 37.5°C and 60% relative humidity and then transferred to a hatching tray. The hatched chicks were grown until sexually mature.

### Testcross and selection of transgenic offspring

The mature individual founder was mated with wild-type quail by placing a male founder with two female wild-type quail or one female founder with one male wild-type quail in a cage. The eggs were numbered, collected daily for one week, and stored in a cold room (13°C) until incubating. The hatched chicks were tagged and reared for 10 days to collect the feather pulp for extracting genomic DNA. The pulp from 1 to 2 feathers was incubated in 300 µl of the cell lysis buffer (200 mM NaCl, 50 mM Tris-Cl, 10 mM EDTA, 1% SDS, pH 8.0) containing proteinase K (0.1 mg/ml, Invitrogen) at 55°C for more than 3 hours. To remove the protein, 100 µl of 7.5 M ammonium acetate was added to the tube, mixed vigorously and then centrifuged at 13,000×*g* for 10 min. The supernatant was transferred to a new tube, and genomic DNA was precipitated by centrifuging after adding and mixing 300 µl of isopropanol. The pellet was washed with 70% ethanol followed by drying and dissolving in TE buffer containing RNase A (10 µg/ml, Qiagen, Valencia, CA, USA). Genomic DNA was used for genotyping PCR with a primer set, LTG0S2-F: 5′- GAGAAGAGACCGAGCCCATC -3′ for forward primer and LT-R: 5′- CGGGCCACAACTCCTCATAA -3′ for reverse primer. The positive offspring were chosen and reconfirmed by PCR with primers, LTG0S2-F and WPRE-R: 5′- AAGGGAGATCCGACTCGTCT -3′for reverse primer. Integration sites of transgene were identified by using the SiteFinding PCR [19]. The SiteFinding PCR was conducted with the primer of SiteFinder-1 and, then, the primary nested PCR was followed by adding the primer mixture of SFP1 and LT-TSP1 (5′- ACTGACAATTCCGTGGTGTT -3′). The PCR product was diluted into 100 µl of distilled water and used as a template for the secondary PCR. The secondary PCR was conducted with primer sets of SFP2 and LT-TSP2: 5′- ACGAGTCGGATCTCCCTTTG -3′ or LT-TSP3: 5′- TGTGCCCGTCTGTTGTGTGA -3′. The bands showing a different size of about 0.3 kb, which is caused by the different position of LT-TSP2 and LT-TSP3, were selected and analyzed to identify the sequences. Nucleotide sequences from each transgenic quail were used to compare the genomic location and sequence homology with chicken at the UCSC Genome Bioinformatics Site (http://genome.ucsc.edu/).

### Tissue collection and weighing

After analyzing exogenous G0S2 expression levels of transgenic lines, two lines, FG1 and FG3, were selected for further study. Five F1 transgenic quail were selected and mated with wild-type quail to produce F2 offspring. Transgenic and non-transgenic male quail were selected by PCR using the primers LTG0S2-F, LT-R, and WPRE-R to detect the transgene, and USP1 and 3 to detect the W-specific DNA fragment [20]. Body weight was measured weekly from hatch until sacrifice at 8 weeks of age. After sacrifice, abdominal fat, subcutaneous fat (Figure S1), left pectoralis muscle, thigh muscle, heart, liver, lung, and kidney tissues were obtained and weighed. Blood samples for the short-term fasting condition were collected after fasting for 4 hours at 7 weeks of age. Other blood samples were collected before sacrifice at 8 weeks of age. After clotting, serum was separated by centrifuging at 1,500×*g* for 15 min. All tissue and serum samples were stored in a -80°C freezer until further study.

### Antibody production and Western blotting

Protein samples were prepared from tissues by grinding with radioimmunoprecipitation assay buffer and centrifuging at 15,000×*g* for 10 min. The G0S2 protein on the membrane was probed with an rabbit anti-chicken G0S2 antibody, which was developed in our laboratory, and an anti-rabbit IgG antibody conjugated with horseradish peroxidase (HRP; Jackson ImmunoResearch, West Grove, PA, USA) was used for detection with an enhanced chemiluminescence system (ECL plus; Amersham, Pittsburgh, PA, USA) and Hyperfilm (Amersham). The specificities of antibodies for quail ATGL and FABP4 proteins were previously demonstrated [21], [22]. After probing the membranes with these ATGL and FABP antibodies, the membranes were incubated with proper secondary antibodies conjugated with HRP. The amount of alpha-tubulin for reference was determined with an anti-alpha-tubulin antibody (12G10; Developmental Studies Hybridoma Bank, Iowa City, IA, USA) and an anti-mouse IgG antibody conjugated with HRP (Cell Signaling Technology, Danvers, MA, USA). The density of the band was determined by using the ImageJ program (NIH, Bethesda, MD, USA).

### Histochemistry and cell size determination

Subcutaneous fat tissues from the non-transgenic and transgenic quail were fixed in 10% neutral buffered formalin (Sigma-Aldrich, St. Louis, MO, USA) for sectioning. All procedures, dehydration, embedding, sectioning, and staining with hematoxylin and eosin were conducted by the Comparative Pathology and Mouse Phenotyping Shared Resource at OSU. Images were taken randomly from sample slides using an AXIO-Vert.A1 optical microscope (Carl Zeiss Microscopy, Thornwood, NY, USA) equipped with an AxioCam MRc5 camera (Carl Zeiss Microscopy) and analyzed by using the ImageJ program to measure the area and cell numbers.

### NEFA assay

The concentration of NEFA in serum was determined by using the NEFA-HR (2) kit that relies on the ACS-ACOD enzymatic method (Wako Diagnostics, Richmond, VA, USA). All procedures were conducted as the manufacturer's protocol. After transferring 5 µl of sera, standards, and deionized water to a 96-well plate, 200 µl of Color Reagent Solution A was added to each well, followed by mixing and incubation at 37°C for 5 min. Then, the first absorbance of each sample was measured at 550 nm for each well. After adding and mixing 100 µl of Color Reagent Solution B to each well, the plate was incubated again at 37°C for 5 min, and the second absorbance was measured at 550 nm for each well. The final absorbance of each sample was obtained by difference. A linear standard curve was generated using the Microsoft Excel program (Microsoft, Redmond, WA, USA), and the NEFA concentration of each sample was calculated based on the standard curve.

### Statistical analysis

Data were presented as mean and standard error of means. Statistical analyses were performed using Student's t-test or analysis of variance. *P* values under 0.05 were considered significant.

## Results

### Generation of transgenic quail expressing exogenous G0S2 in adipose tissue

**Figure 1:**
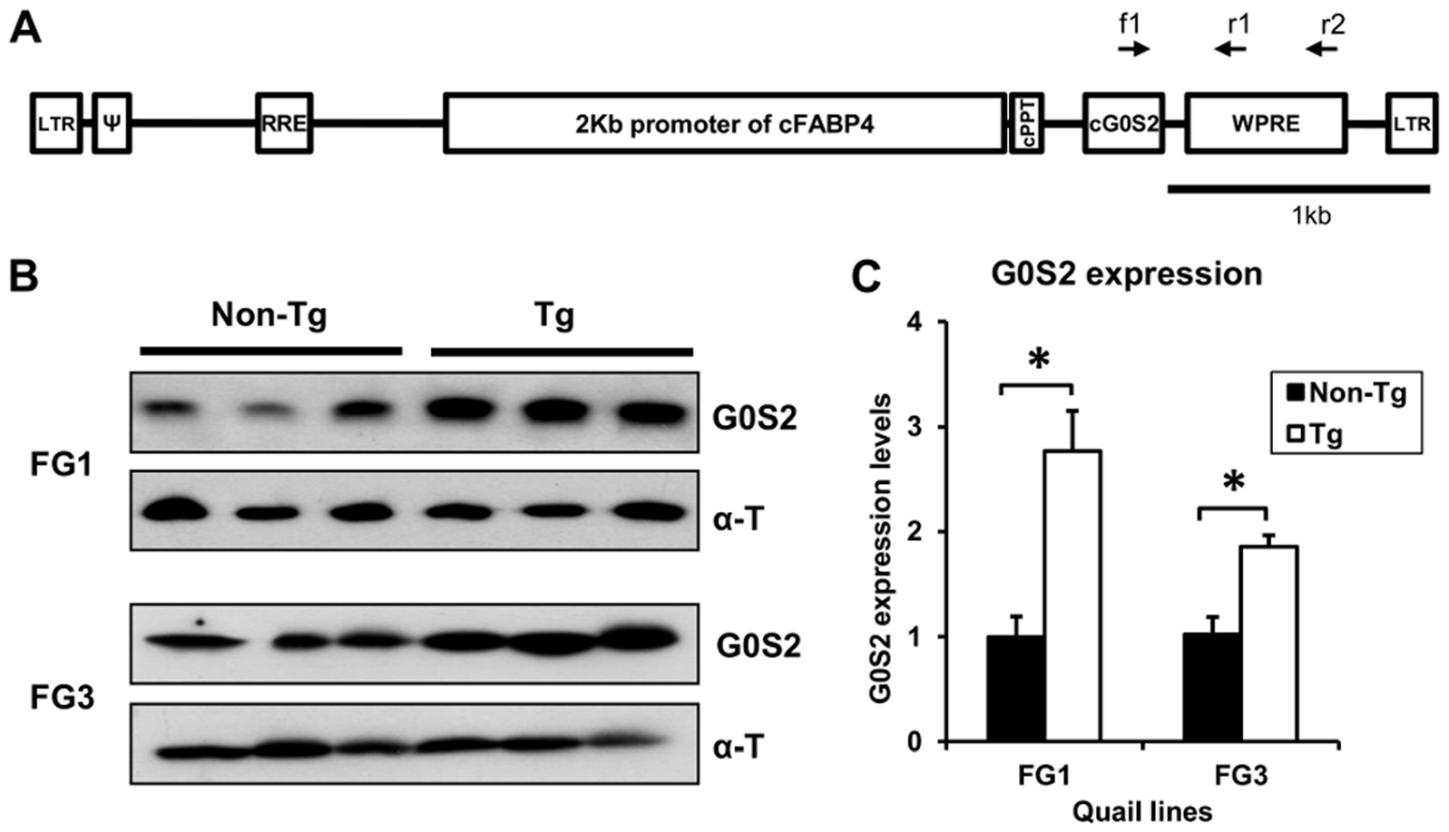
Lentiviral vector construct and its expression in adipose tissues of transgenic quail. (A) Diagram of lentiviral vector used for producing transgenic quail. The vector contains 2 kb promoter of chicken fatty acid binding protein and chicken G0S2 coding DNA sequences. The primers for detecting the transgene are presented as ^f1^ for forward primer (LTG0S2-F) and ^r1^ and ^r2^ for reverse primers (LT-R and WPRE-R, respectively). (B) and (**C**) The expression level of G0S2 protein in adipose tissues of adult transgenic (Tg) and non-transgenic (Non-Tg) quail. Values are represented as mean ± SEM (n = 3). * indicates significance level of *P*<0.05.

The 2 kb promoter of the chicken FABP4 gene and the chicken G0S2 gene were cloned and inserted into a lentiviral vector to express the exogenous G0S2 gene specifically in adipose tissue (Figure 1A). The recombinant lentivirus was injected into 238 quail eggs; 51 founder chicks hatched, and 39 founder quail sexually matured and were crossed with wild-type quail or founders. A total of six transgenic quail lines [FABP4 promoter-G0S2 (FG), FG1 to FG6 lines] were generated from four founders, and the transgene was confirmed by PCR (Figure S2, Table S1). Integration sites of the transgene were analyzed by SiteFinding-PCR (Table S2). Transgenic quail possessed only one copy of the transgene that was located in various integration sites, except for FG3 and FG5 lines, which were generated from the same founder.

Adipose-specific expression of exogenous G0S2 in transgenic quail lines was verified and quantified by western blot analysis using an anti-chicken G0S2 antibody that was recently developed in our laboratory. In wild-type chicken and quail, the G0S2 protein was highly expressed in adipose tissues and negligible in other tissues and organs (Figure S3A), verifying the specificity of the G0S2 antibody in chicken and quail. Among transgenic quail lines, both FG1 and FG3 transgenic quail at embryonic and adult ages expressed 2- to 3-fold more G0S2 proteins in adipose tissues than did non-transgenic quail (Figure 1B and C, and S3C and D). These two transgenic quail lines were used for further investigation of tissue distribution of the G0S2 protein. This abundant expression of G0S2 proteins in adipose tissues in both lines of transgenic quail indicates adipose-specific expression of the exogenous G0S2 protein under the control of the cFABP4 promoter (Figure S3B). Female quail were excluded because of confounding effects caused by their gradual increase in adipose lipolysis to support egg yolk production [23].

### Overexpressed G0S2 has no effect on growth and fat mass *in vivo* under *ad libitum* feeding conditions

**Figure 2:**
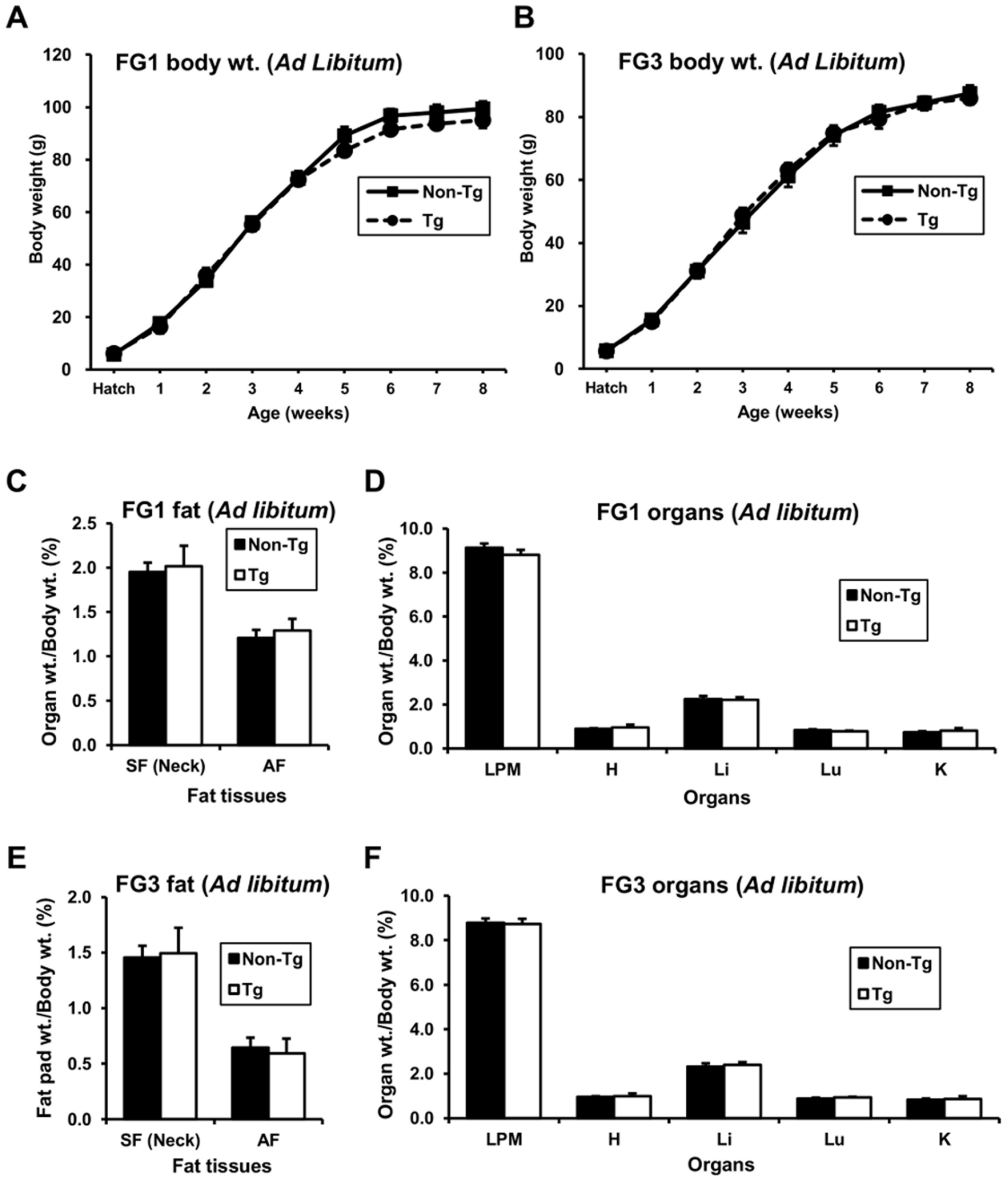
Analysis of growth and organ weights of transgenic and non-transgenic quail fed *ad libitum*. (A and B) Growth curve of FG1 and FG3 lines of transgenic and non-transgenic quail fed *ad libitum*. Body weights of male transgenic and non-transgenic quail were measured every week from hatch to 8 weeks of age. (C and D) Organ weights of FG1 quail fed *ad libitum*. Organ weights were measured and calculated to the percentage of total body weight. There was no difference between transgenic and non-transgenic quail in the tissue and organ weights (*n* = 5). SF: subcutaneous fat, AF: abdominal fat, LPM: left pectoralis muscle, H; heart, Li: liver, Lu: lung, and K: kidney. (E and F) Organ weights of FG3 quail fed *ad libitum*. FG3 did not show a difference in organ weight between transgenic (*n* = 6) and non-transgenic (*n* = 8) quail under the *ad libitum* feeding. Values are represented as mean ± SEM.

In *ad libitum* feeding conditions, there was no difference (*P*>0.05) in body weight between transgenic and non-transgenic quail in either line (Figure 2A and B). The percentages of fat tissue and organ weights relative to body weights were not different between transgenic and non-transgenic quail in both lines (Figure 2C–F). Consequently, overexpressed G0S2 did not affect growth and fat pad weights when sufficient energy was supplied from the diet.

### G0S2 expression prevents loss of fat under feed restriction

**Figure 3:**
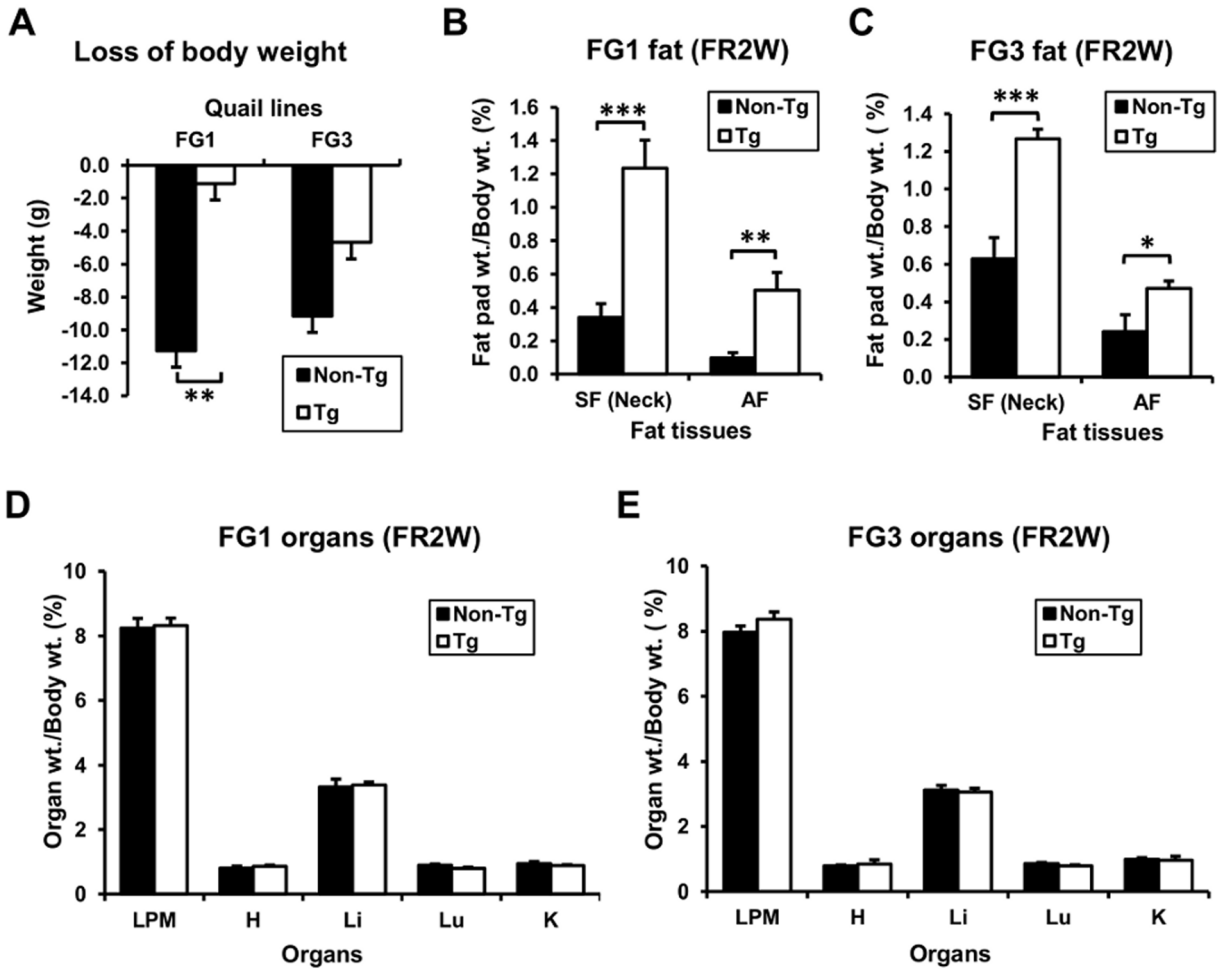
Analysis of body and organ weights of transgenic and non-transgenic quail that underwent feed restriction. (A) Changes of body weights after feed restriction for two weeks. Non-transgenic quail lost more body weight than transgenic quail in both lines. (B and C) Weights of fat pads after feed restriction. Both transgenic quail of FG1 and FG3 had significantly greater fat pads than non-transgenic quail after feed restriction. FR2W: feed restriction for 2 weeks. (D and E) Organ weights after feed restriction. There was no difference in non-adipose organ weights between transgenic and non-transgenic quail after feed restriction. *n* = 6 for each. Values are represented as mean ± SEM. *, **, and *** indicate significance levels of *P*<0.05, *P*<0.01, and *P*<0.001, respectively.

Feed restriction was applied to quail to investigate the effect of G0S2 overexpression under induced lipolytic conditions. The rates of body weight loss by feed restriction were different between transgenic and non-transgenic quail; non-transgenic quail lost about 10 g, whereas FG1 lost only 1g and FG3 lost 5g (Figure 3A). Comparison of adipose tissue weights among these quail revealed that FG1 and FG3 transgenic lines had about 3.6-fold (*P*<0.001) and 2.0-fold (*P*<0.001) more subcutaneous fat around the neck and 5.0-fold (*P*<0.01) and 2.0-fold (*P*<0.05) more abdominal fat, respectively, than non-transgenic quail (Figure 3B and C). Compared with the fat pad weights in the *ad libitum* feeding condition, fat pad weights in non-transgenic quail were reduced 83% and 92% in subcutaneous and abdominal fat tissues, respectively, whereas in FG1 transgenic quail, they were reduced only 39% and 61% in the feed restriction condition. In the FG3 line, fat pad weights in non-transgenic quail were reduced 57% and 63% in subcutaneous and abdominal fat tissues, respectively, but in transgenic quail, they were reduced only 15% and 21%. These results clearly show that all transgenic lines have greater fat pads than non-transgenic quail under feed restriction, indicating that loss of fat during the feed restriction condition was significantly blocked by G0S2 expression in transgenic quail. There was no difference in other organ weights between transgenic and non-transgenic quail (Figure 3D and E), suggesting the effect of G0S2 overexpression could be limited to adipose tissue and not to other tissues in transgenic quail.

### G0S2 prevents adipocyte hypotrophy caused by feed restriction

**Figure 4:**
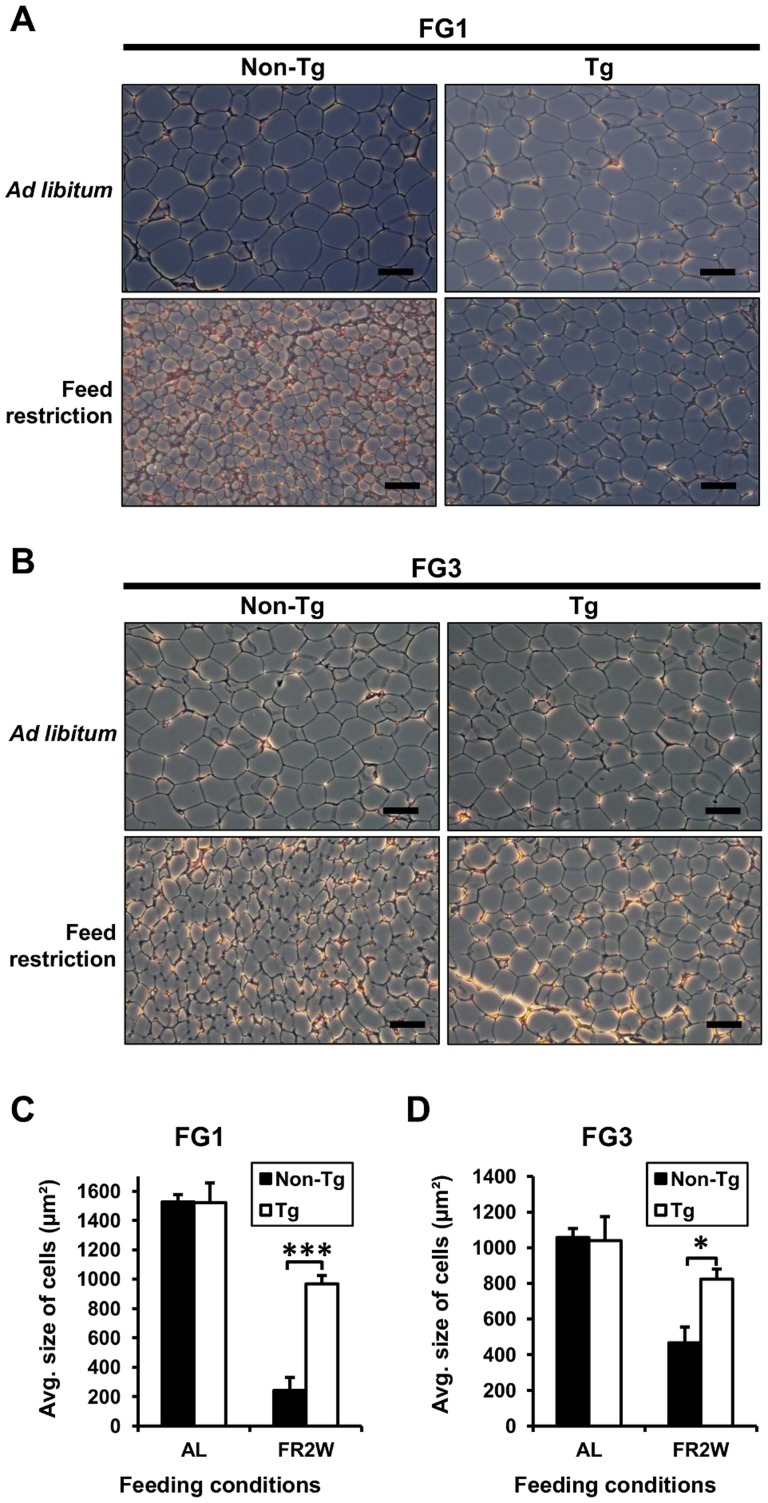
Comparison of fat cell size under the different feeding conditions. (A and B) Histology of fat tissues from transgenic and non-transgenic quail of FG1 and FG3. Fat cell size was almost the same between transgenic and non-transgenic quail in both lines fed *ad libitum*, while it shrank to different levels between them after feed restriction. All scale bars: 50 µm. (C and D) Numerical data of average fat cell size in different feeding conditions. The average fat cell size of non-transgenic quail was significantly smaller than that of transgenic quail only after feed restriction (*n* = 3). AL: *ad libitum* and FR2W: feed restriction for 2 weeks. Values are represented as mean ± SEM. * and *** indicate significance levels of *P*<0.05 and *P*<0.001, respectively.

To investigate whether the differences in fat mass between transgenic quail and non-transgenic quail resulted from changes in fat cell size, the adipose tissues were sectioned, and the size of fat cells was determined. There was no difference in fat cell size between transgenic and non-transgenic quail fed *ad libitum*, as expected, because of the similar fat pad weights. However, the fat cell size of non-transgenic quail was less than that of the transgenic quail in a feed restriction condition (Figure 4A and B). In FG1, the fat cell size of non-transgenic quail was reduced (*P*<0.001) about 4-fold compared to transgenic quail after feed restriction for 2 weeks (Figure 4C). In FG3, the fat cell size of non-transgenic quail was also reduced (*P*<0.05) about 2-fold compared to the transgenic quail after feed restriction (Figure 4D). The rate of reduction in fat cell size in FG1 lines was more severe than in FG3 lines in both transgenic and non-transgenic quail after feed restriction. Therefore, overexpressed G0S2 prevented the adipocyte hypotrophy caused by feed restriction.

### G0S2 inhibits lipolysis in adipose tissue *in vivo*

**Figure 5:**
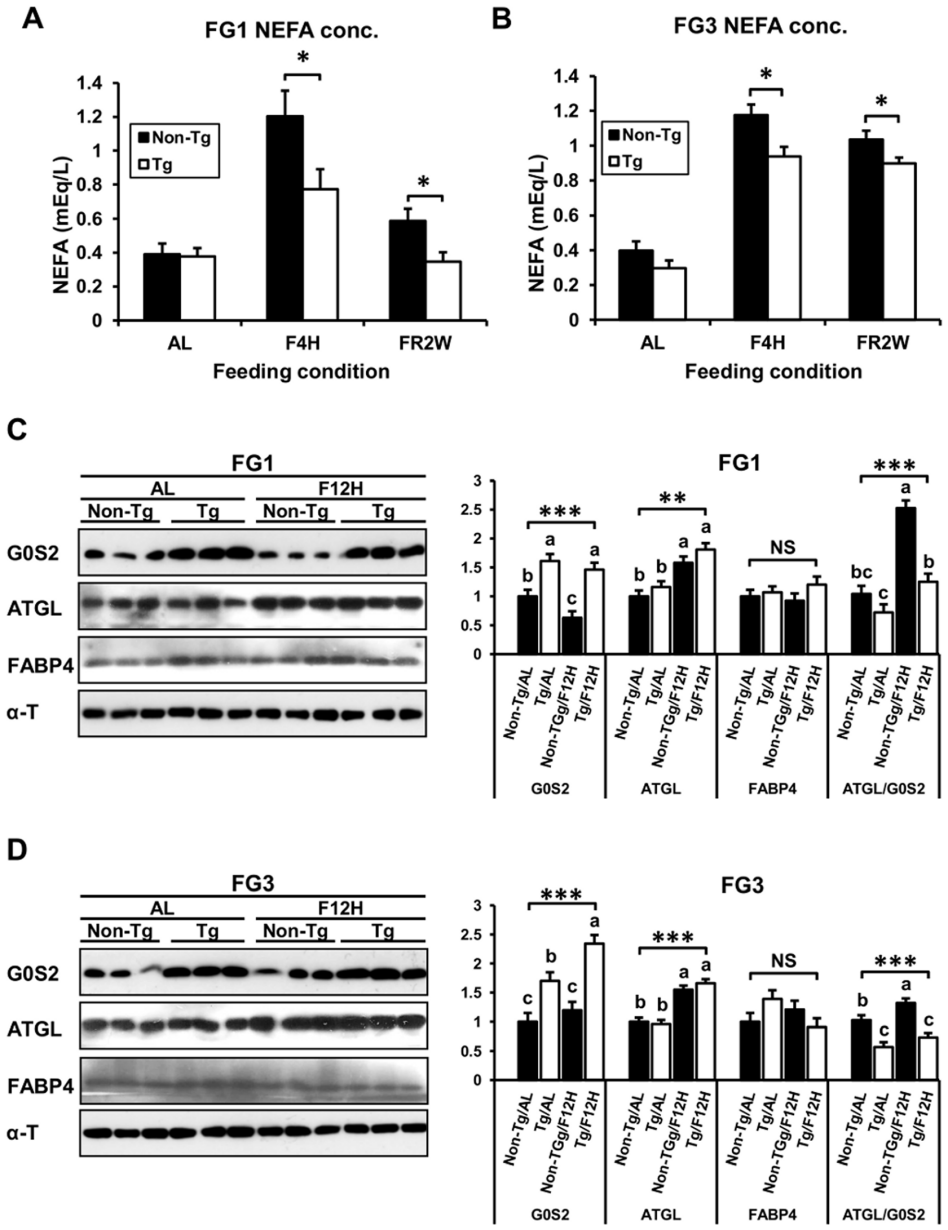
Blood NEFA levels and the expression of G0S2 and ATGL in fat tissues in different feeding conditions. (**A**) and (B) The levels of NEFA in serum were significantly lower in transgenic quail than in non-transgenic quail under the short-term fasting and feed restriction conditions (*n*≥5). AL: *ad libitum*, F4H: fasting for 4 hours, and FR2W: feed restriction for 2 weeks. (C) and (D) The expression of G0S2, ATGL, and FABP4 in fat tissues depending on feeding conditions, *ad libitum* (AL) vs. fasting for 12 hours (F12H). G0S2 was continuously higher in transgenic quail than non-transgenic quail. The expression of ATGL was higher in fasting condition in both transgenic and non-transgenic quail. The ratio of ATGL to G0S2 was highest in non-transgenic quail under the fasting condition. The expression of FABP4 was not significantly variable regardless of feeding condition. Values are represented as mean ± SEM. *, **, and *** indicate significance levels of *P*<0.05, 0.01, and 0.001, respectively. NS means non-significant.

There was no difference in serum NEFA concentration during *ad libitum* feeding between transgenic and non-transgenic quail; however, it was significantly reduced in transgenic quail after short-term fasting and long-term feed restriction, which are considered to induce lipolysis (Figure 5A and B). Transgenic quail of both lines had lower (*P*<0.05) NEFA concentrations in blood than did non-transgenic quail at lipolytic conditions. Analysis of G0S2 and ATGL expression in adipose tissue revealed that G0S2 was expressed higher in transgenic quail than non-transgenic quail during *ad libitum* and 12 hours fasting while the ATGL was up-regulated by 12 hour fasting in both transgenic and non-transgenic quail (Figure 5C and D). Therefore, the ratio of ATGL to G0S2 was higher in non-transgenic quail than transgenic quail, and the ratio was increased more in non-transgenic quail during the fasting condition. The expression of FABP4 was not affected by feeding condition in transgenic and non-transgenic quail of both lines. These data suggest that overexpressed G0S2 in adipose tissues of transgenic quail decreased lipolysis by inhibiting the ATGL during a limited feeding condition.

## Discussion

Recently, Japanese quail have been the focus of many biological studies because of their relatively small size, fast sexual maturation, abundant egg production, and domestication [24]-[28]. In addition, avian transgenesis has a powerful potential in the fields of biology, biomedicine, and agriculture to study embryo development and gene function, to produce therapeutic proteins in the egg, and to generate high-performing and disease-resistant poultry [29]–[31]. Importantly, based on conservation of the metabolic pathways in adipose tissue and similarity to regulation of gene expression in animals, quail were considered a particularly relevant avian model to study functions of target genes in adipose tissue [14], [23], [32].

Although the inhibitory function of G0S2 on ATGL proteins has been recently demonstrated *in vitro*, the anti-lipolytic role of G0S2 in adipose tissue and consequent effect on regulation of fat mass of animals in response to different energy statuses have not been extensively studied *in vivo*. In this study, we generated transgenic quail lines overexpressing the chicken G0S2 gene in adipose tissues under the control of the chicken FABP4 promoter to investigate its role *in vivo*. Adipose-specific expression of transgenes has successfully been achieved using the mouse FABP4 promoter in numerous transgenic mouse models, including ours [33]. Our comparative analysis of FABP4 promoters in mammals revealed conservation of important *cis*-acting elements for the CAAT/enhancer binding protein α (C/EBPα) and peroxisome proliferator-activated receptor γ (PPARγ) binding sites [34]. In the current study, the chicken FABP4 promoter containing two putative C/EBPα and seven PPARγ binding sites successfully expressed the G0S2 protein about 2- to 3-fold greater in adipose tissues of transgenic quail than of non-transgenic quail. The expression of endogenous FABP4 was not affected by feeding conditions and it suggests that the exogenous G0S2 could be expressed stably regardless of feeding conditions.

In chickens, quail, pigs, and humans, expressions of ATGL and G0S2 genes in adipose tissue are dynamically regulated in an inverse relationship at different nutritional conditions such as normal feeding, fasting, and refeeding. Low levels of ATGL and high levels of G0S2 expression at normal feeding and refeeding conditions, and induction of ATGL and reduction of G0S2 expression during the fasting condition are tightly correlated with adipose lipolytic activities and blood levels of NEFA [13], [14], [17]. Under the *ad libitum* feeding condition, overexpression of G0S2 in adipose tissue did not change fat pad weights and fat cell sizes in either line of transgenic quail compared to the control non-transgenic quail. In addition, concentrations of blood NEFA were not different between transgenic and non-transgenic quail, suggesting that lipolytic activities were not affected by overexpression of G0S2 under the *ad libitum* condition. This is different from the result that basal lipolytic activity is reduced by 30 to 40% in fat extracts resulting in about a 2-fold increased fat mass in transgenic mice overexpressing G0S2 compared to wild-type mice [35].The absence of phenotypic responses to overexpression of G0S2 in the transgenic quail could be due to the *ad libitum* feeding condition; a sufficient energy supply from the diet could maintain low ATGL activity during which the inhibitory function of exogenous G0S2 would be minimal. Another plausible explanation based on our previous finding in normal chickens and quail is that high expression of endogenous G0S2 is sufficient to inhibit low amounts of ATGL during the *ad libitum* feeding condition, resulting in blood NEFA maintained at basal levels [14]. Therefore, exogenous G0S2 expression that would be metabolically redundant with high amounts of endogenous G0S2 in the adipose tissue of transgenic quail may not have any additional inhibitory effect on ATGL in an *ad libitum* feeding condition. This hypothesis could explain why G0S2 shows an inhibitory effect on lipolysis under the feed restriction condition while not increasing the fat mass during the *ad libitum* feeding condition.

The above results led us to hypothesize that enhanced ATGL-mediated lipolysis by low energy conditions, such as fasting or feed restriction, is inhibited by overexpression of G0S2. The inhibitory effect of G0S2 on lipolysis *in vivo* was monitored by measuring sequential changes in NEFA release from adipose tissue into blood, in fat cell size, and in adipose tissue weights. In non-transgenic quail, feed restriction for two weeks significantly decreased adipose tissue mass, which is evidently caused by shrinking fat cell size when compared with the cell size before feed restriction. However, greater fat pad weights and fat cell sizes of all transgenic quail lines under the feed restriction status resulted from comparably lower levels of blood NEFA compared with non-transgenic quail lines. In addition, the expression of G0S2 was maintained higher in the adipose tissue of transgenic quail than non-transgenic quail while the ATGL expression was up-regulated, further suggesting the overexpressed G0S2 has the inhibitory role in ATGL activity during the lipolytic conditions. These data clearly indicate that G0S2 overexpression inhibits lipolysis in fat cells resulting in lower release of NEFA to the blood, consequently preventing the loss of TAG stored in lipid droplets that normally occurs by feed restriction. These findings are further supported by the *in vitro* studies showing that overexpression of G0S2 in HeLa cells has an inhibitory effect on lipolysis only under nutrient starvation [3]. Taken together, G0S2 has a significant inhibitory effect mainly on activated ATGL during lipolytic conditions to regulate the rate of TAG hydrolysis.

ATGL-mediated lipolysis could be tightly and inversely regulated by two major modulators: the up-regulation of CGI-58, which is required to activate ATGL in response to the negative energy status, and the induction of G0S2, which is needed to inhibit the activated ATGL during the transition from negative to positive energy status. In the current transgenic quail model, overexpression of G0S2 caused a resistance to loss of fat in a negative energy condition by disrupting the normal temporal regulation of ATGL. Considering the elevated expression of CGI-58 in adipose tissue during the negative energy condition [36], relatively low levels of blood NEFA in the transgenic quail under feed restriction suggest overexpression of G0S2 could dominate the regulation of ATGL by possibly ameliorating or overriding CGI-58 function. This is further supported by the finding that overexpression of G0S2 reduced the lipolytic activity of adipocytes overexpressing ATGL and CGI-58 *in vitro* [3]. Therefore, the treatment to regulate the G0S2 expression could be an ideal approach to change the adiposity in humans and other animals. In addition, it will be interesting to further investigate in the human population whether or not failure of fat loss under a diet program with reduced calorie intake could be related to the G0S2 gene. If so, it would be valuable to determine whether a low release of blood NEFA is attributed to increased G0S2 activity, possibly caused by elevated G0S2 expression or gain-of-function mutations on the G0S2 gene. It is reasonable that fasting or food restriction should be challenged to identify those relations, as the phenotype of G0S2 could not be detected with human individuals who normally access the diet *ad libitum*.

In conclusion, we successfully generated transgenic quail lines overexpressing exogenous G0S2. The excessive G0S2 had an inhibitory effect on lipolysis under feed restriction conditions, but did not enhance fat accumulation under the *ad libitum* feeding condition. Therefore, the G0S2 is a reasonable target gene to treat the adiposity in animals. To our knowledge, this is the first report that investigates gene function by using a transgenic bird and transgenic quail may be a useful animal model in the area of adipocyte biology and obesity research.

## Supporting Information

Figure S1 **Fat pads of quail.** Quail has lots of subcutaneous fats around the neck (a), breast muscle (b and f), and legs (c and e). A big fat pad is located in the abdomen (d). Two major fat pads, subcutaneous fat around the neck (a) and abdominal fat (d), were used for measuring the parameters.
(PDF)

Figure S2 **Detection of transgene in the transgenic quail genome and transgene expression levels.** All transgenic quail were selected by PCR using two primer sets, ^f1^ + ^r1^ (412 bp) and ^f1^ + ^r2^ (798 bp), described in Figure 1A. 1–6: FG1-6 transgenic quail, 7: non-transgenic quail for negative control, and 8: plasmid for positive control.
(PDF)

Figure S3 **Tissue distribution of G0S2 protein in wild-type chicken and quail and in transgenic quail lines.** (A) The level of G0S2 protein in wild-type chicken and quail was investigated in various tissues including subcutaneous fat (SF), abdominal fat (AF), pectoralis muscle (PM), heart (H), liver (Li), lung (Lu), and kidney (K). G0S2 was mainly detectable in fat tissues and barely expressed in heart, liver, and lung. (B) The level of G0S2 protein in various tissues of transgenic quail lines was determined by using Western blot. Total G0S2 protein was mainly detected in adipose tissues. (C) and (D) The relative amounts of G0S2 protein in adipose tissues of transgenic and non-transgenic quail embryos. Two transgenic lines, FG1 and FG3, were selected and investigated to determine the expression level of G0S2 in adipose tissues of embryos at the age of 15 days. Values are represented as mean ± SEM. * and ** indicate significance levels of *P*<0.05 and *P*<0.01, respectively.
(PDF)

Table S1 **The results of testcross for generating transgenic quail.**
(PDF)

Table S2 **Analysis of integration site of transgene on the transgenic quail genome.**
(PDF)
