# Supplementary material for: Inhibition of Lipolysis in the Novel Transgenic Quail Model Overexpressing G0/G1 Switch Gene 2 in the Adipose Tissue during Feed Restriction
Source: PLoS One. 2014 Jun 25;9(6):e100905. doi: 10.1371/journal.pone.0100905 (PMC4071008; doi:10.1371/journal.pone.0100905)
Supplement: Figure S1 — Fat pads of quail. Quail has lots of subcutaneous fats around the neck (a), breast muscle (b and f), and legs (c and e). A big fat pad is located in the abdomen (d). Two major fat pads, subcutaneous fat around the neck (a) and abdominal fat (d), were used for measuring the parameters. (PDF) [file pone.0100905.s001.pdf]

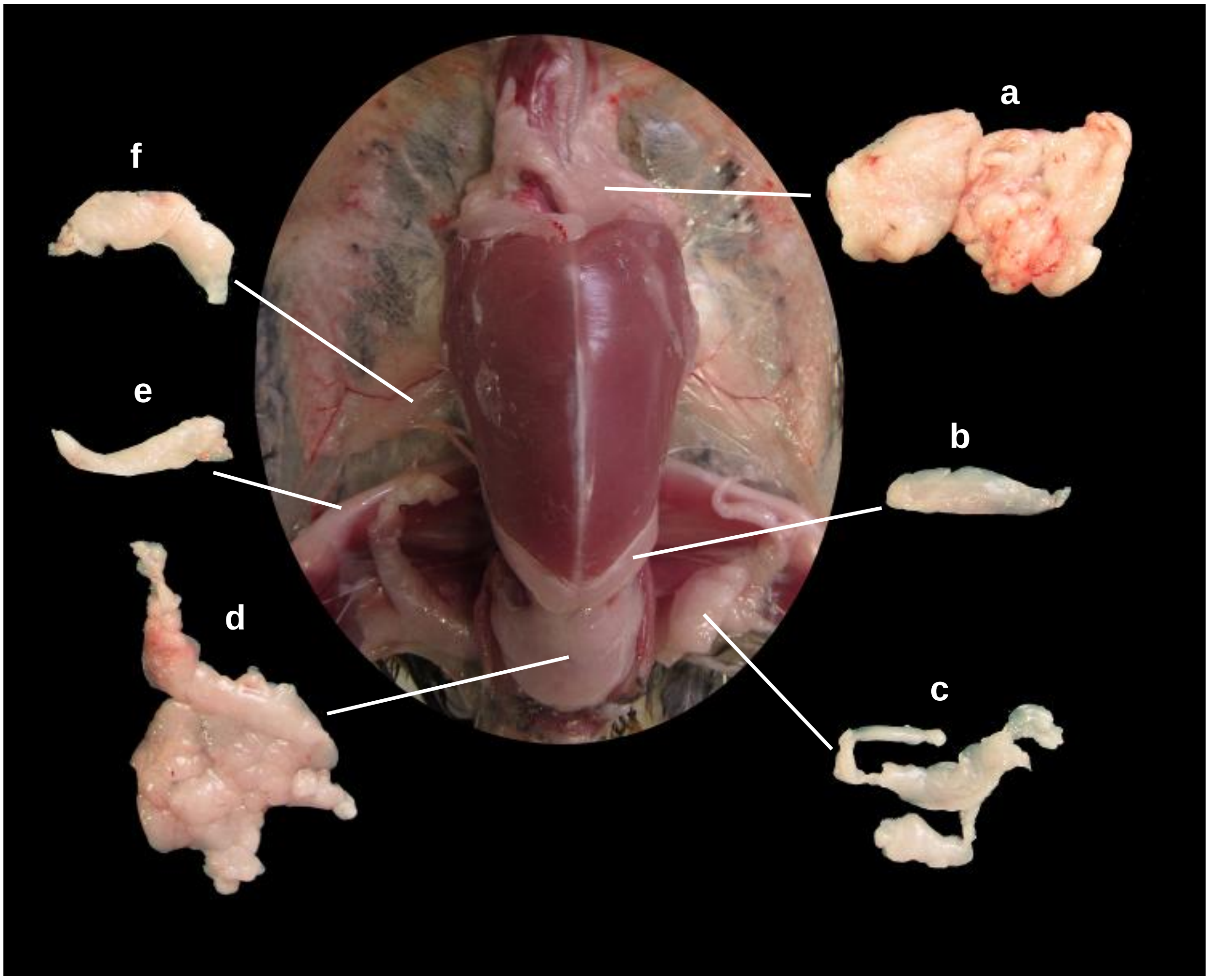

Figure S1. Fat pads of quail. Quail has lots of subcutaneous fats around the neck (a), breast muscle (b and f), and legs (c and e). A big fat pad is located in the abdomen (d). Two major fat pads, subcutaneous fat around the neck (a) and abdominal fat (d), were used for measuring the parameters.
