# Supplementary material for: Inhibition of Lipolysis in the Novel Transgenic Quail Model Overexpressing G0/G1 Switch Gene 2 in the Adipose Tissue during Feed Restriction
Source: PLoS One. 2014 Jun 25;9(6):e100905. doi: 10.1371/journal.pone.0100905 (PMC4071008; doi:10.1371/journal.pone.0100905)
Supplement: Figure S2 — Detection of transgene in the transgenic quail genome and transgene expression levels. All transgenic quail were selected by PCR using two primer sets, f1 + r1 (412 bp) and f1 + r2 (798 bp), described in Figure 1A. 1–6: FG1-6 transgenic quail, 7: non-transgenic quail for negative control, and 8: plasmid for positive control. (PDF) [file pone.0100905.s002.pdf]

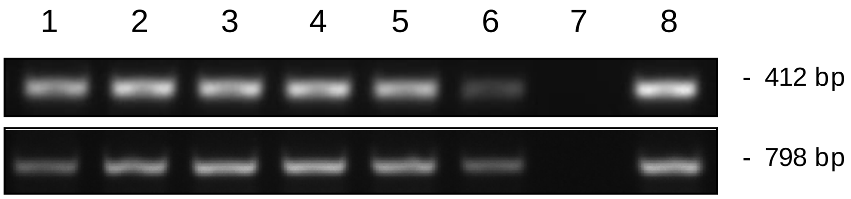

Figure S2. Detection of transgene in the transgenic quail genome and transgene expression levels. All transgenic quail were selected by PCR using two primer sets, ^{f1} + ^{r1} (412 bp) and ^{f1} + ^{r2} (798 bp), described in Figure 1A. 1-6: FG1-6 transgenic quail, 7: non-transgenic quail for negative control, and 8: plasmid for positive control.
