# Supplementary material for: Inhibition of Lipolysis in the Novel Transgenic Quail Model Overexpressing G0/G1 Switch Gene 2 in the Adipose Tissue during Feed Restriction
Source: PLoS One. 2014 Jun 25;9(6):e100905. doi: 10.1371/journal.pone.0100905 (PMC4071008; doi:10.1371/journal.pone.0100905)
Supplement: Figure S3 — Tissue distribution of G0S2 protein in wild-type chicken and quail and in transgenic quail lines. (A) The level of G0S2 protein in wild-type chicken and quail was investigated in various tissues including subcutaneous fat (SF), abdominal fat (AF), pectoralis muscle (PM), heart (H), liver (Li), lung (Lu), and kidney (K). G0S2 was mainly detectable in fat tissues and barely expressed in heart, liver, and lung. (B) The level of G0S2 protein in various tissues of transgenic quail lines was determined by using Western blot. Total G0S2 protein was mainly detected in adipose tissues. (C) and (D) The relative amounts of G0S2 protein in adipose tissues of transgenic and non-transgenic quail embryos. Two transgenic lines, FG1 and FG3, were selected and investigated to determine the expression level of G0S2 in adipose tissues of embryos at the age of 15 days. Values are represented as mean ± SEM. * and ** indicate significance levels of P<0.05 and P<0.01, respectively. (PDF) [file pone.0100905.s003.pdf]

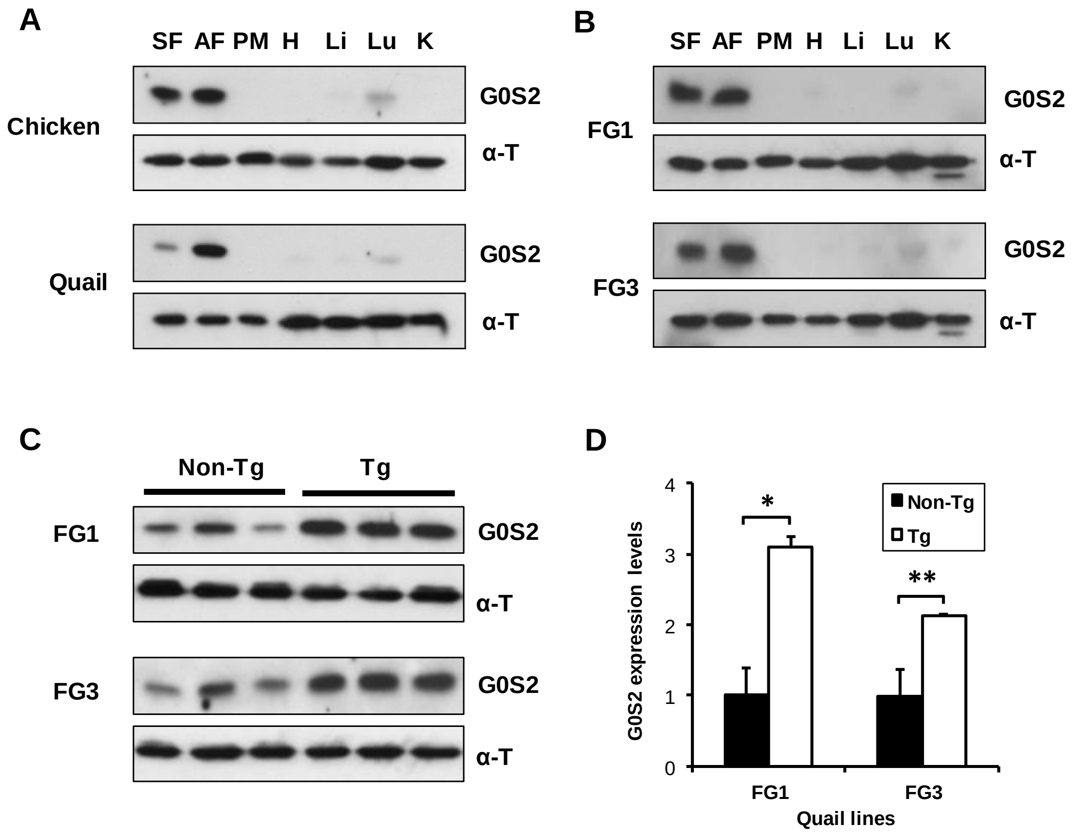

Figure S3. Tissue distribution of G0S2 protein in wild-type chicken and quail and in transgenic quail lines. (A) The level of G0S2 protein in wild-type chicken and quail was investigated in various tissues including subcutaneous fat (SF), abdominal fat (AF), pectoralis muscle (PM), heart (H), liver (Li), lung (Lu), and kidney (K). G0S2 was mainly detectable in fat tissues and barely expressed in heart, liver, and lung. (B) The level of G0S2 protein in various tissues of transgenic quail lines was determined by using Western blot. Total G0S2 protein was mainly detected in adipose tissues. (C) and (D) The relative amounts of G0S2 protein in adipose tissues of transgenic and non-transgenic quail embryos. Two transgenic lines, FG1 and FG3, were selected and investigated to determine the expression level of G0S2 in adipose tissues of embryos at the age of 15 days. Values are represented as mean ± SEM. * and ** indicate significance levels of $P < 0.05$ and $P < 0.01$, respectively.
