# Supplementary material for: Inhibition of Lipolysis in the Novel Transgenic Quail Model Overexpressing G0/G1 Switch Gene 2 in the Adipose Tissue during Feed Restriction
Source: PLoS One. 2014 Jun 25;9(6):e100905. doi: 10.1371/journal.pone.0100905 (PMC4071008; doi:10.1371/journal.pone.0100905)
Supplement: Table S1 — The results of testcross for generating transgenic quail. (PDF) [file pone.0100905.s004.pdf]

Table S1. The results of testcross for generating transgenic quail.

G0 Founder quail		F1 offspring		The ID of transgenic quail (sex)[b]
Male	Female	Total hatched	Transgenic (%)[a]	
2706	Wild type	77	1 (1.3)	FG1 (F)
2712	Wild type	150	2 (1.3)	FG2 (F)
				FG6 (F)
2726	2727	114	2 (1.8)	FG3 (M)
				FG5 (F)
Wild type	2702	145	1 (0.7)	FG4 (F)

[a]The percentage of F1 transgenic offspring among the total hatched chicks is designated in parentheses. [b]The letters in parentheses mean M for male and F for female.
