# Supplementary material for: Inhibition of Lipolysis in the Novel Transgenic Quail Model Overexpressing G0/G1 Switch Gene 2 in the Adipose Tissue during Feed Restriction
Source: PLoS One. 2014 Jun 25;9(6):e100905. doi: 10.1371/journal.pone.0100905 (PMC4071008; doi:10.1371/journal.pone.0100905)
Supplement: Table S2 — Analysis of integration site of transgene on the transgenic quail genome. (PDF) [file pone.0100905.s005.pdf]

Table S2. Analysis of integration site of tansgene on the transgenic quail genome.

Transgenic quail	3'-flanking sequences of the integrated transgene[a]	Comparison to chicken chr.
FG1	ATCTCTAGCAGTTCCTCTGCTCTCTGGTGCAGAAGTC	E22C19W28_E50C23 (+)
FG2	ATCTCTAGCAGGTGTCTAGTACTCCCTAATTCCATCTG	3 (+)
FG3	ATCTCTAGCACCTTTCAATGTGATTAAACGTTAATAAGT	7 (+)
FG4	ATCTCTAGCAGATACATTGGAGTTTCTTAGAATAAGCT	3 (-)
FG5	ATCTCTAGCACCTTTCAATGTGATTAAACGTTAATAAGT	7 (+)
FG6	ATCTCTAGCAACTGACTAACTTTAAAGAATTACCCTAAT	3 (+)

[a]The underlined sequences are the 3' end of lentiviral vector. The 3'-flanking sequence of the integration site was compared to chicken genome sequence by using the UCSC BLAT search.
